# Pathophysiology of mesangial expansion in diabetic nephropathy: mesangial structure, glomerular biomechanics, and biochemical signaling and regulation

**DOI:** 10.1186/s13036-022-00299-4

**Published:** 2022-08-02

**Authors:** Haryana Y. Thomas, Ashlee N. Ford Versypt

**Affiliations:** 1 Department of Chemical and Biological Engineering, University at Buffalo, The State University of New York, Buffalo, NY USA; 2 Institute for Computational and Data Sciences, University at Buffalo, The State University of New York, Buffalo, NY USA

**Keywords:** Diabetic kidney disease, Chronic kidney disease, Mesangium, Renal fibrosis, Mesangial matrix, Mesangial cell, Glomerulus, Extracellular matrix, Collagen accumulation

## Abstract

Diabetic nephropathy, a kidney complication arising from diabetes, is the leading cause of death in diabetic patients. Unabated, the growing epidemic of diabetes is increasing instances of diabetic nephropathy. Although the main causes of diabetic nephropathy have been determined, the mechanisms of their combined effects on cellular and tissue function are not fully established. One of many damages of diabetic nephropathy is the development of fibrosis within the kidneys, termed mesangial expansion. Mesangial expansion is an important structural lesion that is characterized by the aberrant proliferation of mesangial cells and excess production of matrix proteins. Mesangial expansion is involved in the progression of kidney failure in diabetic nephropathy, yet its causes and mechanism of impact on kidney function are not well defined. Here, we review the literature on the causes of mesangial expansion and its impacts on cell and tissue function. We highlight the gaps that still remain and the potential areas where bioengineering studies can bring insight to mesangial expansion in diabetic nephropathy.

## Background

Diabetes is a significant health burden in the US and worldwide. In the year 2015, over 400 million people had diabetes, and the number is expected to rise to over 600 million in the year 2040 [1]. One-third of diabetic patients are estimated to develop diabetic nephropathy, the leading cause of kidney failure [1, 2]. Current therapeutic treatments for diabetic nephropathy are capable of slowing down the progression of kidney damage but do not have the ability to ameliorate or reverse the damage. As such the early diagnosis of diabetic nephropathy is necessary for prevention of kidney failure. However, clinical symptoms of diabetic nephropathy such as proteinuria are only visible in the later stages of diabetic nephropathy making early diagnosis challenging. Consequently, many patients will have to undergo kidney transplant or dialysis, which are expensive and burdensome for the individual as well as for the public health system. As such there is a pressing need to find ways to mitigate the rising burden of diabetic nephropathy.

Mesangial expansion is an important feature in the development of diabetic nephropathy and is characterized by the aberrant proliferation of mesangial cells and matrix protein accumulation in the central region of the glomerulus, the filtration unit of the kidney. Mesangial expansion is frequently used in the clinical diagnosis of diabetic nephropathy and as a marker in the discovery of antagonizing agents [3, 4], in testing the efficacy of a drug [5–7], and in the discovery process of other markers [8]. The quantification of mesangial matrix expansion via biopsy is the gold standard for determining the progression of diabetic kidney damage. Although the exact mechanism of how mesangial expansion causes kidney failure is not well established, mesangial expansion is an important contributor to kidney failure. Thus, understanding the progression of mesangial expansion and determining the mechanism of how mesangial expansion contributes to kidney failure can enable the development of techniques to mitigate and prevent kidney failure.

It is well understood that hyperglycemia is a primary cause for kidney damage in diabetic nephropathy and by extension the primary cause for mesangial expansion. Studies have shown that the stabilization of hyperglycemia to normoglycemia by pancreatic transplant leads to the reversal of structural lesions including mesangial expansion [9]. However, the mechanism by which hyperglycemia leads to mesangial expansion is not well defined. Hyperglycemia has many effects on different parts of the kidney that interact in a complex manner to induce kidney failure. Although mesangial expansion was initially believed to be a result of only hyperglycemia-mediated metabolic derangements in the mesangial cell, many studies have since shown that biomechanical effects due to hypertension and increased glomerular filtration rate (GFR) can also cause mesangial cell dysfunction and thus mediate mesangial expansion. Hypertension and increased GFR can affect the mesangial cell because of the biomechanical linkage of the mesangial cell to the capillaries and to the glomerular basement membrane (GBM), which are exposed to hydrostatic pressures arising from filtration and blood flow.

Thus, in the first section of this review, we explain the biomechanical linkage of the mesangial cell to the GBM and the glomerular capillaries to elucidate the potential mechanisms by which hypertension and increased GFR may mediate mesangial cell dysfunction. In the second section, we highlight the key aspects of the role of hyperglycemia in mediating mesangial cell dysfunction and thus mesangial expansion. Other biochemical and mechanical aspects are also discussed. Since the literature in this field is vast, only representative studies have been selected. In the third section, we review what is known regarding the impacts of mesangial expansion on cell behavior as well as on glomerular function. Additionally, in each of the sections, we highlight gaps where further studies are needed to bring further insight into the complex processes.

## Main text

### Mesangial structure and function

The mesangium was first discovered and described by Zimmerman in 1929 as consisting of mesangial cells and mesangial matrix [10]. Studies by Kimmelsteil and Wilson revealed the importance of this region by discovering the formation of nodules in diabetic patients, the first structural lesion to be identified in the kidney of the diabetic patient [11]. Electron microscope studies have since revealed the structural-functional relationship of this region to the surrounding glomerular tuft. In this section, we examine the structural and functional relationships in the mesangium between the mesangial cell, matrix, and GBM to show that the mesangial cell has functions important to the biomechanical homeostasis of the glomerulus. Mesangial cell dysfunction results in the disruption of the biomechanical homeostasis, which is a concept that is usually ignored in favor of biochemical homeostasis. In order to understand how glomerular failure occurs in diabetic nephropathy, it is imperative to understand the biomechanical structure and function of the mesangial cell and matrix in health and disease.

#### Structure of the mesangial cell

Early investigations uniquely identified the mesangial cell from the rest of the glomerular cells by its central position within the glomerulus [12]. Later, it was found to be associated with matrix synthesis in disease and thus distinguished from neighboring endothelial and visceral epithelial cells [12]. With the advent of the electron microscope, the mesangial cell was further characterized by its unique cytoplasmic structure that enables it to carry out its function [13]. Like most eukaryotic cells, the mesangial cell contains a nucleus, mitochondria, a golgi apparatus, and endoplasmic reticulum; however, its golgi apparatus and endoplasmic reticulum are underdeveloped, and its cytoplasm sparsely populated [13, 14].

In the quiescent state, the mesangial cell does not have a robust protein or matrix synthesis system [14]. In addition to the quiescent phenotype, the mesangial cell exhibits activated and hypertrophied phenotypes. The quiescent phenotype is characterized by a stellate cell shape, a low proliferation rate, and a sparse cytoplasm. The activated phenotype is characterized by an elongated cell shape, high proliferation rate, alpha smooth muscle actin (*α*-sma) expression, and synthesis of interstitial collagens (Table 1) [14–17]. The activated cell phenotype is usually associated with diseased conditions where increased proliferation rates and synthesis of interstitial collagens are observed [15]. The activated mesangial cell is reminiscent of myofibroblasts in other tissues, which exhibit characteristics of both smooth muscle cells and fibroblasts such as the expression the of *α*-sma, responsiveness to vasoactive agents, and production of interstitial collagens [15, 17]. The hypertrophied phenotype is characterized by a lowered proliferation rate, a high expression of *α*-sma, and a polygonal cell shape [14, 18, 19].

**Table 1:** Markers and mediators of different mesangial cell phenotypes. MC: mesangial cell, FCS: fetal calf serum, TGF- *β*: transforming growth factor *β*, PDGF- *β*: platelet derived growth factor *β*, Coll: collagen. Note: there is disagreement in the literature regarding the expression level of *α*-sma by differerent mesangial cell phenotypes; here, we show that through two rows in the table for the corresponding expression levels

Attribute	Quiescent MC	Activated MC	Hypertrophied MC	Sources
Proliferation rate	Low	High	Low	[14, 15, 17]
Cell shape	Stellate	Elongated	Polygonal	[18]
*α*-sma expression	None	Low	High	[18, 19]
*α*-sma expression	None	High	-	[16, 17]
Key mediators	-	Presence of FCS, PDGF- *β*	TGF- *β*, Absence of FCS	[18, 19]
Associated matrix proteins	Coll IV	Coll I, Fibronectin	Coll IV	[19]

**Fig. 1:**
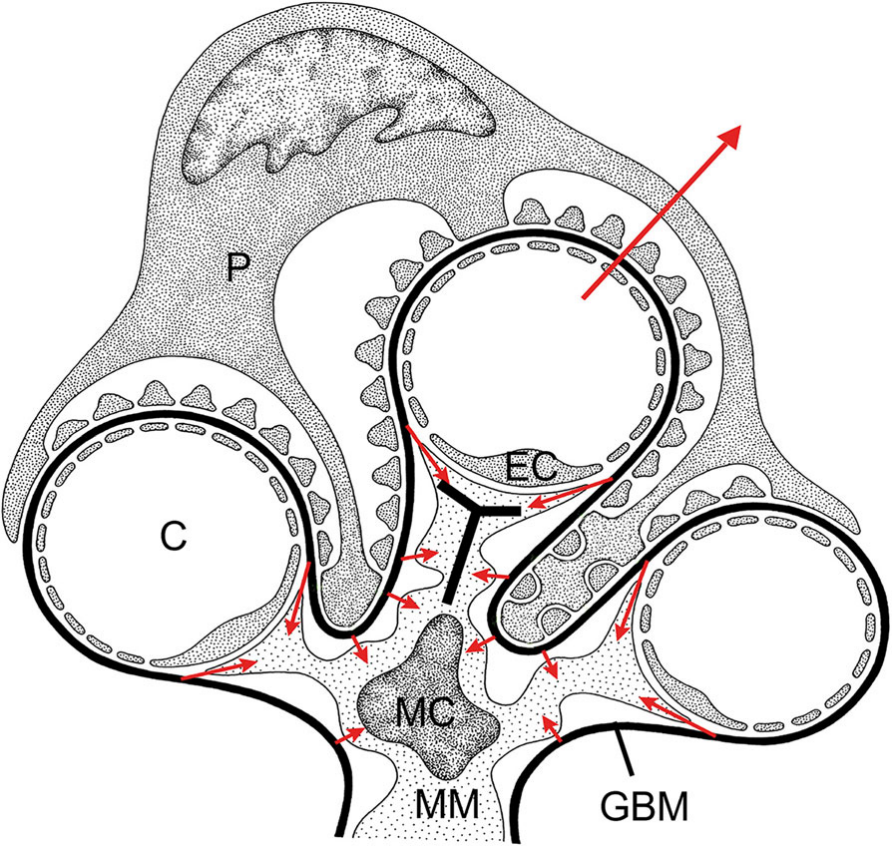
Mesangial cell structure with its processes extending out to multiple capillaries. MC: mesangial cell, MM: mesangial matrix, P: podocytes, EC: endothelial cells, C: capillaries, GBM: glomerular basement membrane. The small arrows indicate the mesangial cell/GBM connections that oppose the hydrostatic pressures shown by long red arrow. The T-shaped marking indicates the mesangial cell process where the body of the T-shaped marking represents the axis of the mesangial processes, and the head of the T-shaped marking indicates the mesangial angles. The mesangial cell occupies a majority of the mesangium and is closely attached to the GBM directly by microfibrillar attachments or indirectly to the GBM via the mesangial matrix. The T-shaped mesangial processes are firmly embedded in between the capillaries and the GBM and enable the mesangial cell to maintain glomerular structure. Modified after [75] with permission

The most distinct features of mesangial cells are the cytoplasmic processes that extend out from the core of each mesangial cell towards the capillary lumen (Fig. 1) [12–14, 17]. These processes contain cytoplasmic fibrils that are 7-10 nm in diameter [17] and enable the cell and its cytoplasmic processes to attach to the surrounding matrix and GBM. The ends of these cytoplasmic processes branch out around the capillary walls creating wide contact areas between the mesangial processes and endothelial cells [13]. These processes, however, are not attached to the endothelial membrane nor are they connected by specialized intermembrane connections [13]. Instead, these branched processes wedged in between the GBM and the endothelial membrane are tightly attached to the GBM by microfibrils or indirectly connected through the mesangial matrix [13].

The mesangial processes enable the mesangial cell to carry out its most important function—the stabilization of the glomerular capillary structure. Without such stabilization, the glomerulus would collapse and fail to carry out filtration. The discovery of these processes also fueled a longstanding debate about the role of mesangial cell contractility in regulating the filtration rate of the glomerulus [17, 20–22]. Although, less debated today, the hypothesis that mesangial cells and their processes can regulate filtration rate has not been fully discarded.

#### Structure and composition of the mesangial matrix

Early investigators of the mesangial region had the notion that the mesangial matrix was just an extension of the glomerular basement membrane surrounding the capillaries [12]. This mistake was understandable given that both the basement membrane and the mesangial matrix have similar composition. However, further studies revealed key differences between the mesangial matrix and basement membrane that correspond to the structural-functional relationships of both the basement membrane and the matrix.

The glomerular basement membrane is mainly composed of high-density collagen IV, laminin, and the proteoglycan heparan sulfate [23]. In contrast, the mesangial matrix is composed of low-density collagen IV, various proteoglycans including heparan sulfate, high concentration of fibronectin, and a low concentration of laminin [17].

These differences in composition result in significant differences in the properties and behavior of basement membrane and mesangial matrix, which enable the two to carry out their specialized functions. The high density of collagen IV in basement membranes provides structural integrity and elasticity to the GBM to be able to withstand the high capillary pressures. In contrast, the low-density collagen and the presence of water-binding proteoglycans [17] enable the mesangial matrix to oppose both expansile forces and compressive forces. The high concentration of glycoproteins such as fibronectin enable the binding of the cell to the structural components of the matrix [13, 24].

The mesangial matrix is gel-like [12] and contains extensive structural elements that are important for its function. Electron microscopy studies describe a fine meshwork of non-collagenous microfibrils (11-15 nm diameter) indirectly attaching the basement membrane to the mesangial cell processes [13]. The indirect attachments are augmented by direct attachments between the GBM and the mesangial cell [25]. The extensive structural elements and the intimate connection between the GBM and the mesangial cell enable the maintenance of the glomerular structure [22]. Without such frequent close attachments, hydrostatic pressures across the perimesangial GBM cause mesangial expansion [25].

Although the mesangial matrix is not considered a basement membrane, its composition indicates that it is more like a basement membrane than an interstitial matrix. It does not contain the major components found in interstitial matrices such as collagen I and collagen III, but rather consists of collagen IV, the main component of basement membrane matrix [17]. In diabetic nephropathy, however, the composition of the matrix is altered where even collagen III is observed [26].

#### Function of the mesangial cell and matrix

An important mesangial cell function is the maintenance of glomerular structure. Unlike the bulk of the vasculature, glomerular capillaries do not have a basement membrane that completely encircles the capillary lumen (Fig. 1), as it is blocked by a portion of the capillary lumen that is exposed to the mesangium. In other vasculature, the expansile forces due to blood flow are counterbalanced by wall tension provided by the vascular wall and the active contractile elements within the vascular wall [22]. However, due to the incomplete wrapping of the GBM around the capillary lumen, the GBM cannot develop wall tension independently. Likewise, the endothelial lining of the capillary lumen is incapable of providing wall tension because it is very thin and highly fenestrated [13]. Thus, the mesangial cell acts as an anchor for the GBM to be able to provide wall tension to resist the distending forces arising within the capillary [22]. This mechanism is reliant on the presence of strong connections between the mesangial cell, its matrix, and the GBM [22, 27]. Failure of these connections lead to capillary ballooning and bulging [12, 22, 27] and displacement of capillary towards urinary space leading to podocyte effacement or lesion formation [27]. Perfusion of glomeruli with high pressures breaks mesangial and GBM connections, eventually leading to capillary dilation [22]. Hypertension and hypertrophy, dysfunctions commonly associated with diabetic patients [28], can contribute to the mechanical failure of the mesangium [22].

Hypertension can also increase the stress experienced by the mesangial cell/GBM connections along the axis of the mesangial cell processes. Although, these mesangial cell/GBM connections are weak and susceptible to failure, they occur frequently enough to be able to oppose the distending forces of the hydrostatic pressure in the mesangial region [25]. However, increases in hydrostatic pressures can break these mesangial cell/GBM connections resulting in mesangial expansion [22, 25].

One of the hypothesized functions of mesangial cells was the regulation of GFR [21] via a reduction in capillary diameter [17]. In vivo studies of GFR indicated that the infusion of vasoactive agents led to a 50 percent reduction in the GFR [21]. The hypothesis was further supported by in vitro experiments that showed that mesangial cells contracted when exposed to vasoactive agents such as angiotensin II (Ang II) [29]. Electron microscope studies provided the mechanisms by which mesangial cell contraction could lead to a reduction in capillary diameter and thus GFR [13]. However, further electron microscope studies revealed that mesangial cell contraction could only reduce capillary diameter by 10 percent, which could not account for the 50 percent reduction in GFR that was observed in vivo [22]. Additionally, the in vivo studies showed significant increase in the efferent arteriolar resistance, which could have mediated the decrease in GFR [21]. It is well known today that the vasoconstriction and vasodilation of the glomerular arterioles mediated through vasoactive agents regulate glomerular hemodynamics, which directly impacts GFR [30]. Although the regulation of GFR is mainly mediated by arteriolar constriction and dilation, the involvement of mesangial cells in the regulation of GFR has not been completely discarded and awaits full elucidation [22].

Overall, the structure of the mesangial cell in relation to the GBM and the capillaries indicates that the mesangial cell’s functions are biomechanical in nature. As such in disease conditions such as diabetic nephropathy, dysfunction of the mesangial cell can lead to a failure of the mesangial cell to carry out its biomechanical functions. However, the extent to which the mesangial cell’s biomechanical functions are altered and how they, in turn, affect overall glomerular function is not well known.

### Progression of mesangial expansion in diabetic nephropathy

Mesangial expansion is one of the key structural changes observed in the glomerulus 5-7 years after the onset of diabetes in humans [31, 32]. Prior to mesangial expansion, GBM thickening and kidney hypertrophy are the earliest observed structural changes [28, 32–34]. However, metabolic and hemodynamic alterations such as hyperglycemia and hypertension generally precede and occur concurrently with the observed structural alterations [35].

Mesangial expansion occurs as a consequence of aberrant mesangial cell proliferation, mesangial matrix accumulation, and hypertrophy induced by the diabetic state. It is now well established that metabolic and hemodynamic alterations within the kidney are the primary causes of kidney failure in diabetes and by extension the causes of abnormal structural changes such as mesangial expansion. In vivo and in vitro models of the diabetic state have elucidated more clearly how metabolic and hemodynamic alterations lead to mesangial expansion. The results indicate that the biomechanical link of the mesangial cell to the glomerular capillaries enable hemodynamic alterations to play a role in the progression of mesangial expansion and that hemodynamic alterations exert their influence through metabolic derangements in the mesangial cell. Thus, the two are closely intertwined and augment each other in the progression of mesangial expansion.

#### The role of hyperglycemia in mediating matrix protein accumulation

Matrix protein accumulation is characteristic of mesangial expansion. Immunohistochemical studies showed that native collagens such as collagen IV, V, and VI and non-native collagens such as collagen III accumulated in the mesangium during diabetic nephropathy [26]. In vitro studies showed that the incubation of mesangial cells in high glucose concentrations resulted in the accumulation of matrix components such as collagen I, collagen IV, fibronectin, and laminin (Table 2) [36–39]. Further studies elucidated that either the increased synthesis of collagen [37, 40] or the inhibition of collagen degradation [38] led to collagen accumulation. It is possible that short-term accumulation of collagen is mediated by increased synthesis and that long-term accumulation is mediated by the inhibition of collagen degradation. A thorough investigation elucidated that high glucose conditions did not cause the diabetic milieu, but that the increased uptake of glucose was the cause of the matrix accumulation, which was mediated by the upregulation of Glut-1 receptors [40].

**Table 2:** The role of hyperglycemia in matrix protein accumulation in in vitro studies. The effect of hyperglycemia on upregulation of matrix proteins is reduced after long periods of incubation. TGF- *β*: transforming growth factor - *β*, N.S.: not significant, -: indicates no data. A single arrow pointing upwards indicates a statistically significant increase in the respective protein expression, and a double arrow indicates a substantially larger increase in protein expression relative to control

Time frame	2 weeks	8 weeks	1 week	4 weeks	6 weeks	24 hrs	24 hrs	48 hrs
	[38]		**[**37**]**	[36]		**[**40**]**	[41]	
Collagen IV expression	*↑↑*	*↑*	*↑*	*↑↑*	*↑*	*↑*	-	-
Collagen I expression	*↑↑*	*↑*	-	-	-	*↑*	-	-
Fibronectin expression	-	-	*↑*	*↑↑*	*↑*	*↑*	N.S.	*↑*
Laminin expression	-	-	*↑*	*↑↑*	*↑*	*↑*	-	-
Protease expression	*↑↑*	*↑*	N.S.	-	-	*↑*	-	-
TGF- *β* expression	*↑↑*	*↑*	-	-	-	-	*↑*	*↑↑*

Hyperglycemia-induced matrix accumulation is mediated by TGF- *β*. TGF- *β* is significantly upregulated during the diabetic milieu in in vitro studies [38, 41], diabetic animal models [42, 43], and human diabetes [43] and has been repeatedly implicated in mediating matrix accumulation [38, 41, 42]. TGF- *β*’s role in matrix accumulation [44, 45], the intracellular signaling pathways involved [46–48], and its role as a potent profibrotic agent in other fibrotic diseases [49] have been extensively reviewed.

Ang II also mediates matrix accumulation indirectly through TGF- *β* upregulation [50]. The activation of the renin-angiotensin system in the kidney stimulates an increase in Ang II [51]. The effect of Ang II on mesangial cells is two-fold: directly through Ang II receptors on mesangial cells and indirectly through changes in glomerular capillary pressure, which also impacts mesangial cell behavior. Angiotensin converting enzyme (ACE) inhibitors have been successful in ameliorating glomerular injury due to their ability to reduce hypertension and thus mediate the multifold effects of Ang II on mesangial cells [51].

The signaling pathways and molecular mechanisms involved in diabetic nephropathy that lead to extracellular matrix (ECM) accumulation, proliferation, and hypertrophy have been extensively reviewed elsewhere [52–56]. Briefly, there are multiple intracellular pathways that are activated during DN. Key extracellular mediators such as hyperglycemia, advanced glycation end products, and reactive oxygen species (ROS), which are upregulated during DN, are responsible for activating the various intracellular pathways involved in ECM accumulation [52, 53]. One of the main modes of activation is through the upregulation of TGF- *β*, which can activate SMAD and non-SMAD pathways that lead to the accumulation of ECM proteins. In the SMAD pathway, TGF- *β* activates its kinase receptors which activate the SMAD pathways via phosphorylation and leads to the production of ECM proteins. Non-SMAD pathways such as mitogen-activated protein kinases (MAPK), extracellular signal-regulated kinase/c-JUN N-terminal kinase (ERK/JNK), and phosphatidylinositol 3 kinase (PI3K) can also be activated by TGF- *β* to result in the increased accumulation of ECM proteins [53]. The Janus kinase (JAK)-signal transducer and activator of transcription (STAT) pathway has also been implicated in mediating the accumulation of ECM proteins by regulating the proliferation of ECM-producing cells [53]. The JAK/STAT pathway is also involved in hypertrophy of mesangial cells, production of TGF- *β*, and production of ECM proteins in hyperglycemic conditions [52, 54]. Nuclear factor *κ*B (NF- *κ*B) is another key pathway involved in the progression of DN. The NF- *κ*B pathway mediates the production of inflammatory molecules that contribute to the accumulation of ECM proteins [52, 54]. Similar to the other pathways, NF- *κ*B is also activated by key extracellular signals such as hyperglycemia, cytokines, Ang II, mechanical stress, and ROS [52, 54]. ROS also stimulates fibronectin production via inhibitor of myogenic differentiation family isoform a [57].

Calcium operated signaling pathways have also been implicated in regulating the production of ECM proteins during DN. Activation of store-operated Ca2+ entry (SOCE) has been shown to negatively regulate the high glucose and TGF- *β* mediated increase in collagen IV and fibronectin production by mesangial cells [58]. However, it has also been shown to promote hyperglycemia-mediated upregulation of fibronectin and collagen [59] through the activation of downstream calcium signaling pathways. The negative regulation of ECM proteins by SOCE has been shown to be through the inhibition of SMAD 1 [60] and the TGF- *β*/SMAD 3 pathway [61] and also through the regulation of inflammatory molecules such as interleukin-6 [62].

#### The role of mechanical stimuli in matrix component accumulation

Hemodynamic changes that occur during diabetes, such as hypertension, increase the mechanical forces experienced by mesangial cells. These mechanical forces have been found to stimulate matrix component accumulation. Increase in glomerular pressures in perfused glomeruli have been shown to lead to significant increases in mesangial cell stretching (12 percent increase) [63]. Studies have also found that in diabetes, disruption of glomerular pressure autoregulation results in mesangial cell elongation of 10 percent compared to the normal 4 percent elongation experienced in health [30]. In vitro studies showed that mesangial cell stretching results in the upregulation of matrix components collagen IV, collagen I, fibronectin, and laminin [63–65]. Unlike mesangial cells stimulated by hyperglycemia, mechanically stimulated mesangial cells exhibit increased synthesis of collagen IV rather than collagen I, mimicking in vivo observations of diabetes [63].

Hyperglycemic and mechanical stimuli are highly intertwined because of the similarities in the mechanism and the intermediate agents by which matrix protein accumulation is induced. Similar to hyperglycemia, mechanically induced matrix protein accumulation is mediated by TGF- *β*. Its latent form is upregulated during mesangial cell stretching and mediates matrix protein accumulation [65, 66]. Additionally, the mechanism by which TGF- *β* induces matrix protein accumulation is via increased glucose flux into the cell through the upregulation of Glut-1 receptors, which is the same mechanism as hyperglycemia-induced matrix protein accumulation [30]. Combining the hyperglycemic stimulus with mesangial cell stretching augments the collagen accumulation in an additive manner further indicating that the two stimuli work in concert to bring about mesangial expansion [64].

#### The role of hyperglycemia in mediating proliferation and hypertrophy of mesangial cells

Aberrant proliferation of mesangial cells is a characteristic of many diseases that affect the kidney including diabetes [15]. The role of hyperglycemia in mediating mesangial cell proliferation has been studied in vitro and in vivo, and this hyperglycemia-induced proliferation is transient [16, 67]. However, these non-monotonic transients are not observed in human diabetic patients [33]. Mesangial cells cultured in high glucose conditions showed an initial proliferation phase in the first 48 hours followed by an inhibited phase where proliferation rate of mesangial cells was decreased [67]. Inhibition of the initial proliferation was mediated by TGF- *β* [67]. The long-term inhibition of mesangial cell proliferation in the in vitro diabetic model has been corroborated by others [40]. In vivo studies within diabetic rat models have also verified this transient proliferation of mesangial cells [16]. An in vivo study within the streptozotocin (STZ) induced rat showed that significant proliferation of mesangial cells occurred within the first 3 days after the induction of diabetes but then decreased over the following 30 day period eventually reaching normal levels [16]. In other studies, following inhibition of mesangial cell proliferation in the diabetic milieu, mesangial cells underwent cellular hypertrophy where cellular mass increases without cell proliferation [28, 40].

The role of mechanical stimuli on mesangial cell proliferation and hypertrophy have not been fully elucidated. However, we can gain some insight from smooth muscle cells and fibroblast cells, which behave similarly and respond to mechanical cues in a similar manner. These cells have been shown to respond to stress by increased proliferation, contraction, and collagen I and III synthesis [68]. The mechanism by which stress induces these responses in smooth muscle cells and fibroblasts is via integrins to an intracellular pathway or indirectly through the activation of biochemical signals such as Ang II, TGF- *β*, and PDGF, which are known to mediate cellular proliferation, matrix synthesis, and cellular contraction [68].

The time scale involved in the upregulation of matrix component accumulation when mesangial cells are cultured in high glucose conditions is shown in Table 2. The time scale for the upregulation of matrix proteins starts as early as the first day, but significant accumulation only occurs after weeks of incubation [36, 38, 41]. This data also shows that long periods of incubation lead to lower levels of upregulation compared to controls indicating that matrix proteins actually increasingly degrade as the period of incubation increases. This is opposite to what is observed in diabetic animal models where longer periods of the diabetic state result in greater differences in collagen accumulation compared to controls (Table 3) [16, 42, 43].

**Table 3:** Collagen accumulation, proliferation, and TGF- *β* expression in the STZ rat diabetes model. In contrast to in vitro data, upregulation of matrix proteins increases after long periods of incubation. TGF- *β*: transforming growth factor - *β*, N.S.: not significant, -: indicates no data. The arrows indicate the magnitude of change relative to controls. A single arrow pointing upwards indicates a statistically significant increase in the respective protein expression, and a double arrow indicates a substantially larger increase in protein expression relative to control

Time frame	3 days	30 days	3 days	7-14 days	6 weeks	15 weeks
	[16]		[42]		[43]	
Proliferation rate	*↑*	*↓*	-	-	-	-
Collagen accumulation	*↑*	*↑↑*	*↑*	*↑↑*	-	-
TGF- *β* expression	N.S.	*↑*	N.S.	*↑*	*↑*	*↑↑*

Additionally, collagen I is expressed in control cultures and expressed in higher levels than collagen IV in stimulated mesangial cells. This is unusual because quiescent mesangial cells do not produce collagen I. Additionally, collagen IV should be increasingly more accumulated than collagen I in stimulated cells. In vitro models need to be improved so that they more accurately mimick observations from diabetic animal models. Diabetic animal models also need to be improved because the proliferation of mesangial cells is transient, which is not the case in human diabetic patients [33].

### Impact of mesangial expansion on cell and tissue function

**Fig. 2:**
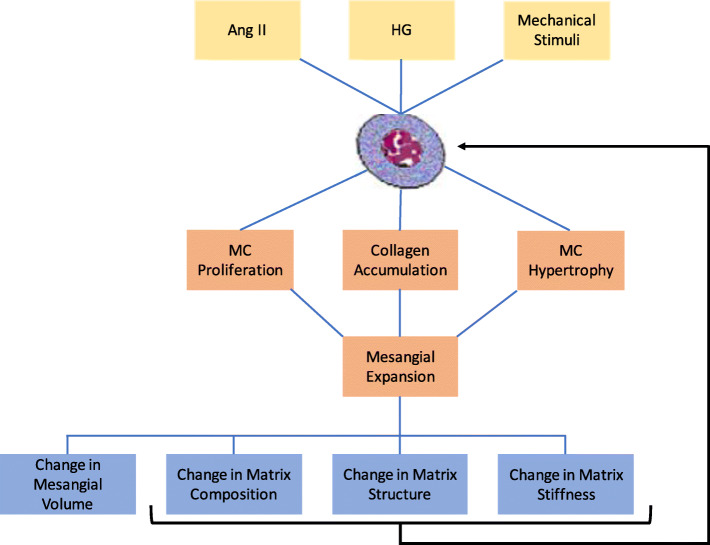
A representation of the multiscale process that governs mesangial expansion from the molecular level (yellow) to the cellular level (orange) and tissue level (blue). Interactions between these different scales are important, especially the feedback influence of changes in mesangial matrix structure, composition, and stiffness on mesangial cell behavior. Ang II: angiotensin II, HG: hyperglycemia

The cause and effect relationship of mesangial expansion to cell and glomerular function occurs across multiple length scales. Figure 2 is a representation of the different scales involved in the progression of mesangial expansion. At the molecular and cellular levels, biochemicals stimulate the mesangial cell phenotype, proliferation rate, and matrix synthesis. These changes, in turn, have an impact on the structure and function of the mesangial tissue such as changes in matrix composition, structure, and volume. Since the structure of the mesangial cell, matrix, and GBM plays a very important role in the function of the glomerulus, changes in the tissue composition and structure have a significant impact on glomerular function. Alterations in mesangial matrix composition, volume, and structure have a feedback effect on cell behavior further exacerbating mesangial expansion. In the following section, we discuss the interactions between the mesangial cell and the mesangial matrix in relation to mesangial expansion.

#### The impact of mesangial matrix structure and composition on mesangial cell behavior

During mesangial expansion, the composition of the mesangial matrix varies with the different stages of mesangial expansion. Early stages of mesangial expansion are associated with accumulation of native collagens such as collagen IV, and late stages are associated with the accumulation of interstitial collagens such as collagen I and III [17, 26]. The variation in matrix composition and structure of the mesangial matrix can lead to the exacerbation of mesangial expansion in a feedback manner.

In vitro modulation of the mesangial matrix impacts mesangial cell behavior, which contributes to the progression of mesangial expansion [69–72]. Rat mesangial cells cultured on collagen I matrix exhibited higher proliferation and migration rates than mesangial cells cultured on basement membrane matrix (Table 4) [69]. This behavior of rat mesangial cells has also been observed in human mesangial cells [70]. The increase in migration and proliferation rates indicate the activation of the mesangial cells, which is supported by mesangial cell elongation, another indicator of mesangial cell activation [69, 70]. Although the expression of alpha-smooth muscle actin was not investigated in those studies, it can be seen from the increased proliferation, migration, and elongation that culturing mesangial cells on collagen I matrix leads to the activation of mesangial cells. However, culturing mesangial cells on basement membrane matrix results in the maintenance of the quiescent phenotype [69], which is characterized by the absence of proliferation, migration, and cell elongation. Since the activated mesangial cell is a phenotype expressed in disease [15] and a phenotype associated with aberrant proliferation in diabetes [16], mesangial cell activation by the mesangial matrix results in the exacerbation of mesangial expansion.

**Table 4:** The role of matrix structure and composition in regulating mesangial cell behavior. Bm: basement membrane. Elongated cell shape indicates activated phenotype of mesangial cell, while a stellate shape would have indicated quiescent phenotype. Extended cell shape description is an ambiguous descriptor. Diseased matrix is an aligned matrix scaffold, whereas a normal matrix is a nonaligned matrix scaffold

Matrix type	Diseased matrix [71]	Collagen I matrix [69]	Collagen I matrix [70]
Control	Normal matrix	Bm matrix	Bm matrix
Cell shape	elongated	extended	elongated
Proliferation rate	*↑*	*↑*	*↑*
Migration rate	-	*↑*	*↑*
*α*-sma expression	*↑*	-	-
Collagen IV expression	*↓*	-	-
Collagen I expression	*↑*	-	-
TGF- *β* expression	*↑*	-	-

In addition to the higher proliferation and migration rates exhibited by activated mesangial cells, these cells also produce interstitial collagens that are not native to the mesangial matrix, specifically collagens I and III [15]. In the quiescent state, mesangial cells produce native collagens such as collagen IV [15]. Type IV collagen forms sheet-like matrices, whereas type I collagen forms crosslinked matrices [17]. Differences in matrix structure have been shown to cause mesangial cell activation [71], a phenotype that contributes to the exacerbation of mesangial expansion. One study used a nanopatterned scaffold that mimicked abnormal matrix structures and found characteristics of activated mesangial cells such as alpha-smooth muscle actin expression, higher proliferation rates, and cellular elongation (Table 4) [71]. In this state, the mesangial cells produced increasing amounts of collagen I, TGF- *β*, and fibronectin but not collagen IV or laminin, which are the major components of the normal mesangial matrix (Table 4) [71]. Thus, abnormal matrix structures led to mesangial cell activation, which resulted in the production of non-native collagens and growth factors implicated in the progression of mesangial expansion [71].

The mechanism by which cell-matrix interactions modulate cell behavior is through integrins. A study showed that mesangial cells cultured on basement membrane matrix had higher cell viability than mesangial cells cultured on fibronectin, collagen I, or plastic [72]. The extent of cell viability was dictated by the level of mesangial cell adhesion to the substrate mediated by *β*-1 integrin [72]. This finding indicates that integrin-mediated cellular adhesion regulates cell viability. In the same way, integrins could mediate other mesangial cell behaviors, such as proliferation, migration, and activation, which have been shown to be sensitive to the composition and structure of the substrate [69–71]. In fibroblasts and smooth muscle cells, integrins transduce mechanical signals for cell proliferation, matrix protein metabolism, and cellular contraction [68]. Since the effect of matrix properties on cell behavior is transduced through integrins, one would expect integrins and matrix properties to have similar effects on mesangial cells. However, the presence of specific integrins has been found to ameliorate mesangial expansion [3].

**Fig. 3:**
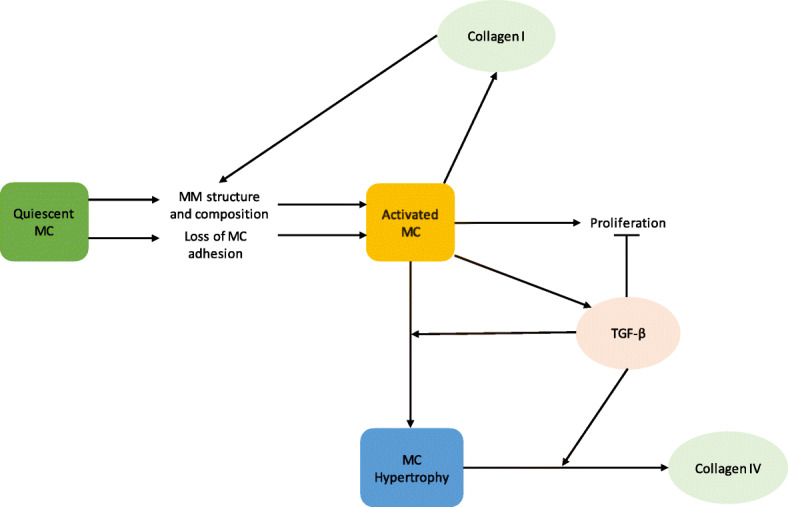
The role of the mesangial matrix in regulating mesangial cell phenotype, proliferation, and collagen accumulation. Structural and compositional alterations to the mesangial matrix cause the activation of the mesangial cell, which enhances proliferation and collagen I production. TGF- *β* production by the activated mesangial cell causes mesangial cell hypertrophy and prevention of mesangial cell proliferation. MC: mesangial cell, MM: mesangial matrix, TGF- *β*: transforming growth factor beta. Pointed arrows represent enhancement, and flathead arrows indicate inhibition

The mesangial matrix is a dynamic environment that is actively in communication with the mesangial cell. A network representation of the interaction between the mesangial matrix and mesangial cell phenotypes is shown in Fig. 3. The structure and composition of the mesangial matrix mediates mesangial cell activation in opposition to TGF- *β*, which suppresses mesangial cell activation (Fig. 3).

The mechanism by which matrix structure mediates mesangial cell activation is not fully clear. However, it is known that mesangial matrix composition activates the mesangial cell via reduced adherence of the mesangial cell to the mesangial matrix. The phenotype that the mesangial cell exhibits dictates the rates of proliferation, type and quantity of matrix synthesized and degraded, and the types of biochemicals released, which all impact the progression of mesangial expansion. Experimental studies are needed that characterize the specific phenotypes exhibited when the mesangial cell is cultured on different matrices. Additionally, the mechanisms by which mesangial matrix structure and composition modulate mesangial cell phenotype and the extent to which the mesangial matrix impacts mesangial cell phenotype also need to be elucidated.

#### The impact of matrix stiffness on mesangial cell behavior

In other well studied tissues, mechanical signaling from the matrix contributes to the regulation of various cell behaviors such as proliferation, migration, differentiation, apoptosis, and cell-matrix interactions [73]. In cancer, matrix stiffness has been shown to exacerbate tumor progression in a feedback manner [74]. Matrix stiffness also regulates the phenotype of vascular smooth muscle cells [73] and the motility of cells [73]. When cells are on soft substrates, they exhibit apoptosis, growth arrest, few focal adhesions, and are not fibrogenic, but cells cultured on stiff substrates are proliferative, migratory, extended, and fibrogenic [73]. Mesangial cells fit this pattern because when they are activated, they are increasingly proliferative, produce higher levels of interstitial collagen, are more extended, and produce increasing amounts of adhesion components such as fibronectin [14–17]. Mechanical stimuli could play a role in the mesangial matrix-mediated modulation of mesangial cell behavior. Especially since mesangial expansion is a fibrotic process, it is possible that the change in mechanical properties of the mesangial matrix contributes to the exacerbation of mesangial expansion in a feedback manner. However, the changes in mechanical properties of the mesangial matrix during diabetes are not known and thus need to be characterized before conclusive remarks can be made about the full role of mesangial matrix mechanics on mesangial expansion.

#### The role of mesangial expansion in glomerular failure

Mesangial expansion in diabetic nephropathy has significant impact on mesangial cell function and glomerular function. It has been proposed that mesangial expansion can lead to the reduction in GFR that is commonly observed in diabetic patients [31, 32]. Mesangial expansion was found to have a strong inverse correlation with capillary surface area and GFR of patients at different stages of DN [32, 33]. Meanwhile, GBM thickness was not strongly correlated with GFR [32, 33]. Additionally, a correlation between mesangial expansion and proteinuria was found, but no correlation was found between GBM thickness and proteinuria [32, 33]. These results indicated that mesangial expansion is an important structural change, even more so than the GBM thickening, in the progression of diabetic nephropathy.

It has, however, been observed in some cases that mesangial expansion does not lead to glomerular failure [32]. Even though patients have mesangial expansion, some did not have proteinuria or changes in their GFR, and it was hypothesized that a glomerulus can accommodate a certain amount of mesangial expansion without any loss in glomerular function [32]. However, once the mesangium expands beyond the accommodative capability of the glomerulus, the mesangium will expand into the capillary space causing a reduction of the filtration area and thus a reduction in the filtration rate [32]. Electron microscopy studies show extensive folding of the GBM in the mesangial region [13]. The extent of GBM folding dictates the extent to which a glomerulus can accommodate mesangial expansion by unfolding of the GBM folds. Differences in the level of GBM folding in patients could account for the heterogeneity of the impact of mesangial expansion on glomerular function.

Mesangial expansion affects the entire glomerulus by occluding the capillaries; forcing podocytes together resulting in podocyte effacement and loss; and disrupting mesangial cell, GBM, and glomerular structure. All of these processes affect cellular and glomerular function and could contribute to clinically observed symptoms of kidney failure such as lowered GFR and proteinuria. However, quantitative studies are required to elucidate the exact mechanisms by which mesangial expansion contributes to kidney failure and to test the theories for the heterogeneous impact of mesangial expansion on kidney function. Bioengineering approaches from tissue engineering, quantitative systems biology, and in vivo imaging hold promise for tackling the myriad of challenges in understanding and developing therapeutics for the complex interacting processes involved in mesangial expansion during diabetic nephropathy.

## Conclusions

In this review, we survey the causes and impact of mesangial expansion in diabetic nephropathy in relation to the biomechanical structure and function of the mesangial cell, the metabolic and hemodynamic causes of mesangial expansion, and the impacts of mesangial expansion on cellular and glomerular function. The biomechanical structure and function of the mesangial cell indicates that the mesangial cell’s biomechanical function is susceptible to failure. This susceptibility to failure could be contributing to the exacerbation of mesangial expansion. Especially important structural features are the mesangial cell/GBM connections whose failure can cause dysregulation of glomerular structure, prevent adherence of mesangial cell to basement membrane, and disable the mesangial cell from opposing the forces arising from hydrostatic pressures. The metabolic causes that contribute to mesangial expansion are well established through the use of in vitro and in vivo diabetic models. These studies have shown that hyperglycemia can induce mesangial matrix accumulation and mesangial cell hypertrophy. However, these diabetic models still need to be improved to provide accurate representation of processes occurring in mesangial expansion. Mesangial expansion has an impact on cellular and glomerular function. The changes in the mesangial matrix that arise during mesangial expansion affect mesangial cell behavior in a feedback manner. The change in structure and composition of the matrix can result in the expression of the activated mesangial cell phenotype leading to the exacerbation of mesangial expansion.

## Data Availability

Not applicable.

## Abbreviations

*α*-sma: alpha smooth muscle actin
Ang II: angiotensin II
ACE: angiotensin converting enzyme
Bm: basement membrane
C: capillaries
Coll: collagen
EC: endothelial cells
ECM: extracellular matrix
FCS: fetal calf serum
GBM: glomerular basement membrane
GFR: glomerular filtration rate
HG: hyperglycemia
MC: mesangial cell
MM: mesangial matrix
N.S.: not significant
PDGF: platelet-derived growth factor
P: podocytes
ROS: reactive oxygen species
STZ: streptozotocin
TGF- *β*: transforming growth factor beta
